# Causality Investigation between Gut Microbiome and Sleep-Related Traits: A Bidirectional Two-Sample Mendelian Randomization Study

**DOI:** 10.3390/genes15060769

**Published:** 2024-06-12

**Authors:** Mingxia Zhai, Weichen Song, Zhe Liu, Wenxiang Cai, Guan Ning Lin

**Affiliations:** 1 Shanghai Mental Health Center, Shanghai Jiao Tong University School of Medicine, School of Biomedical Engineering, Shanghai Jiao Tong University, Shanghai 200240, China; 2 Shanghai Key Laboratory of Psychotic Disorders, Shanghai Jiao Tong University, Shanghai 200240, China

**Keywords:** sleep-related traits, gut microbiome, bacterial pathways, Mendelian randomization, causal relationship

## Abstract

Recent research has highlighted associations between sleep and microbial taxa and pathways. However, the causal effect of these associations remains unknown. To investigate this, we performed a bidirectional two-sample Mendelian randomization (MR) analysis using summary statistics of genome-wide association studies (GWAS) from 412 gut microbiome traits (N = 7738) and GWAS studies from seven sleep-associated traits (N = 345,552 to 386,577). We employed multiple MR methods to assess causality, with Inverse Variance Weighted (IVW) as the primary method, alongside a Bonferroni correction ((*p* < 2.4 × 10^−4^) to determine significant causal associations. We further applied Cochran’s Q statistical analysis, MR-Egger intercept, and Mendelian randomization pleiotropy residual sum and outlier (MR-PRESSO) for heterogeneity and pleiotropy assessment. IVW estimates revealed 79 potential causal effects of microbial taxa and pathways on sleep-related traits and 45 inverse causal relationships, with over half related to pathways, emphasizing their significance. The results revealed two significant causal associations: genetically determined relative abundance of pentose phosphate decreased sleep duration (*p* = 9.00 × 10^−5^), and genetically determined increase in fatty acid level increased the ease of getting up in the morning (*p* = 8.06 × 10^−5^). Sensitivity analyses, including heterogeneity and pleiotropy tests, as well as a leave-one-out analysis of single nucleotide polymorphisms, confirmed the robustness of these relationships. This study explores the potential causal relationships between sleep and microbial taxa and pathways, offering novel insights into their complex interplay.

## 1. Introduction

Sleep is a reversible state characterized by an increased perceptual threshold to external stimuli, resulting in a lack of responsiveness to the environment [1]. It is an indispensable process for the human body, occupying one-third of human life [2] and playing a crucial role in maintaining physical and mental health. However, sleep disorders are becoming increasingly prevalent in society, affecting 37–58% of adults [3] and causing significant harm to cognition and physical performance [4,5]. Research indicates a close association between sleep and various diseases, including cardiovascular disease [6], diabetes [7], mental disorders [8], and even mortality [9]. Additionally, a study on Polish patients with temporomandibular disorders found that poor sleep quality is associated with increased muscle pain and reduced life satisfaction [10]. However, the mechanisms underlying sleep disorders are not fully understood. Early studies have primarily focused on the role of the central nervous system in the control and potential disruption of sleep [11]. The involvement of peripheral organs, such as the gut, in sleep regulation is yet to be fully understood.

The gut microbiome, a highly complex microbial community, may directly or indirectly participate in sleep regulation through the microbiome–gut–brain axis [12]. Several studies have reported associations between the gut microbiome and sleep. For instance, microbial taxa from the *Christensenellaceae* family have been observed to correlate positively with rapid eye movement (REM) sleep and negatively with continuous glucose levels, potentially indicating a relationship between microbiota and metabolic processes during sleep [13]. In a comparative study, antibiotic-induced microbiota-depleted mice exhibited noticeable differences in sleep/wake architecture compared to control mice [14]. Gut microbes and their metabolites have been implicated in mediating the ameliorative effects of melatonin on cognitive impairment induced by sleep deprivation [15]. Conversely, sleep deprivation has been found to significantly reduce the α-diversity in the gut flora, leading to decreased gut microbiome diversity and metabolic dysbiosis [16].

However, the presence of potential confounding factors such as diet, environment, and age reduces the reliability of these experimental results. Furthermore, due to ethical constraints, many randomized controlled trials are difficult to conduct, limiting the inference of causal relationships between gut microbiota and sleep.

Mendelian randomization is a method for estimating the causal effect of an exposure on an outcome using genetic variants as instrumental variables [17] and addresses some of these challenges. These genetic variants, determined at conception and randomly allocated among individuals, are less susceptible to confounding or reverse causality [18]. This approach allows for distinguishing between correlation and causation.

In this study, a bidirectional two-sample MR analysis was employed to assess the causal relationship between 412 microbial taxa and pathways and 7 sleep-related traits. Notably, the gut microbiome data in this study are derived from the Dutch Microbiome Project (DMP) cohort [19], which utilizes metagenomic sequencing for microbes, enabling species-level identification. Additionally, this includes the identification of bacterial pathway abundances, facilitating a comprehensive exploration of the relationship between gut microbiota and sleep.

## 2. Materials and Methods

### 2.1. Description of Study Design

**Figure 1:**
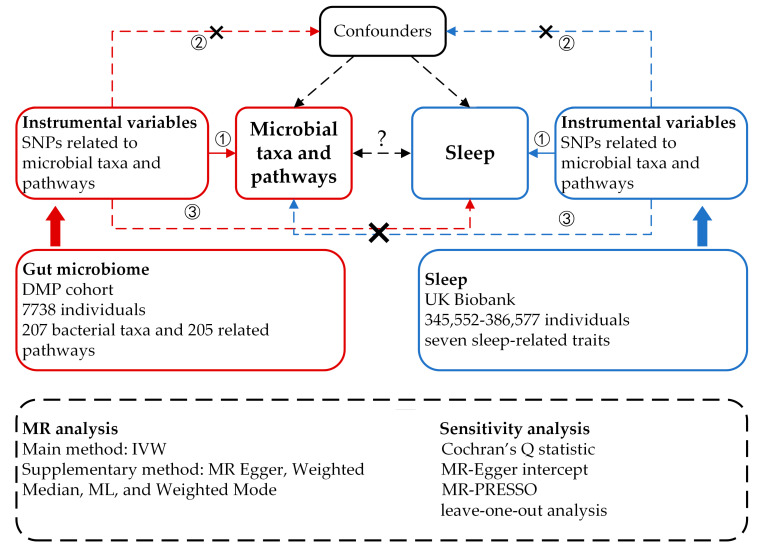
Study design of the bidirectional two-sample MR analysis on the associations of gut microbiome and sleep. The numbers ①, ②, and ③ represent the three key assumptions of MR mentioned in Section 2.1. The arrows represent directional influences between elements, the X represents the absence of a direct effect and the ? represents unknown interactions between elements.

As shown in Figure 1, we implemented a bidirectional two-sample MR design to explore potential causal relationships between 412 microbial taxa and pathways and 7 sleep-related traits. For MR-derived causal estimates to be valid, three key assumptions, as outlined in previous studies [20,21], must be satisfied: (1) A strong association exists between the genetic variants and the exposure. (2) The genetic variants are independent of any confounding factors that could simultaneously influence the exposure and outcome. (3) The genetic variants affect the outcome risk solely through the exposure, not other pathways.

In the forward MR analyses, we considered 207 taxa and 205 pathways as the exposures, with each sleep phenotype serving as the outcome. Conversely, in the reverse MR analyses, each sleep phenotype was treated as the exposure, with the 207 taxa and 205 pathways as the outcomes. To ensure robustness and reliability in our causal inferences, we employed a diverse array of MR methodologies. These included MR Egger, Weighted Median, IVW, Weighted Mode, and Maximum Likelihood (ML) approaches. Additionally, we conducted several sensitivity analyses to further validate our findings, encompassing heterogeneity tests, pleiotropy tests, and leave-one-out analyses.

### 2.2. Data Source

The genome-wide association studies (GWAS) summary statistics data for the human gut microbiome were sourced from the Dutch Microbiome Project’s GWAS dataset. This extensive project undertook metagenomic sequencing of fecal samples from 7738 participants in The Netherlands, aged 8–84, aiming to elucidate the relationship between human genetic variations and the gut microbiome. This cohort stands out from many existing ones that utilize 16S RNA sequencing by enabling bacterial identification at the species level and identifying the abundance of bacterial pathways. In total, our analysis encompassed 207 taxonomies, including 5 phyla, 10 classes, 13 orders, 26 families, 48 genera, and 105 species, alongside 205 bacterial pathways [19].

Concerning sleep-associated phenotypes, we utilized GWAS summary data from the study by Jansen et al. [22], which were derived from the UK Biobank. This expansive, population-based cohort includes over 500,000 participants aged 40–69 [22]. The sleep data were obtained from self-reported sleep questionnaires completed during the initial UK Biobank research visit between 2006 and 2010. These questionnaires covered seven sleep-related items: insomnia, morningness, sleep duration, ease of getting up in the morning, daytime napping, daytime sleepiness, and snoring [23]. The participant counts for these seven phenotypes ranged from 345,552 to 386,577. Further details about these two GWAS summary datasets are available in Supplementary File S1: Table S7. The study cohorts for exposure and outcome are both comprised of European individuals, ensuring consistency in the analyzed populations.

### 2.3. Instrumental Variable Selection

In the forward MR analysis, single nucleotide polymorphisms (SNPs) associated with 207 taxa and 205 pathways representing microbial composition were selected using a locus-wide significance threshold (*p* < 1.0 × 10^−5^) [24]. To address the issue of strong linkage disequilibrium, we applied stringent criteria, including an R² cut-off of less than 0.001 and a clumping window of 10,000 kb, referencing the 1000 Genomes European data as our standard. SNPs present in the exposure data but absent in the outcome data were excluded [25]. Additionally, we eliminated SNPs with a minor allele frequency (MAF) below 0.01. To mitigate the risk of weak instrumental bias, SNPs with an F-statistic (calculated as $F=\frac{R^{2}(N\;-\;k\;-\;1)}{k(1\;-\;R^{2})}$) lower than 10 were also removed. The above SNP filtering ensures a strong correlation between SNPs and exposure. Additionally, we investigated each instrumental variable using ieu open gwas to avoid its association with potential confounders (e.g., socioeconomic status, smoking, alcohol use, obesity, and stress [26]). To meet the assumption that the instrument is associated with the outcome only through exposure, we excluded SNPs closely associated with the outcome. For palindromic SNPs, forward-strand alleles were inferred using allele frequency data.

For the reverse MR analysis, instrumental variables (IVs) for the seven sleep-related traits were selected using a threshold of *p* < 5.0 × 10^−8^, considering the large sample size. The screening conditions for this selection were consistent with those employed in the forward MR analysis.

### 2.4. Mendelian Randomization Analysis

We employed multiple statistical models to estimate the bidirectional causal relationships between gut microbiota and sleep-related traits. These included MR Egger, Weighted Median, IVW, ML, and Weighted Mode, with IVW serving as the principal method. IVW integrates Wald ratio estimates for each SNP through a meta-analysis approach, providing a comprehensive effect estimate of exposure on outcome. This method is efficient when all genetic variants are valid instrumental variables IVs [27], but it can be biased in the presence of pleiotropic genetic variables. The ML method, similar to IVW, assumes no heterogeneity and the absence of horizontal pleiotropy in the IVs [28]. When these conditions are met, ML results are generally consistent with IVW. The Weighted Median approach, which infers causality from the median of weighted empirical density functions derived from individual SNP effect estimates, is robust even if up to half of the genetic variants are invalid [28]. Weighted Mode groups SNPs based on the similarity of their causal effects and estimates the causal effect using the cluster with the most SNPs [29]. It offers an unbiased estimate, provided that the SNPs within the largest cluster are valid.

Considering multiple hypothesis testing, we applied a Bonferroni correction for the *p*-value in the IVW method. Specifically, the threshold for the 207 taxa and 205 pathways was set at 2.4 × 10^−4^ (0.05/207, 0.05/205) [30,31]. After correction, results with a *p*-value less than the Bonferroni-corrected thresholds and simultaneously *p* < 0.05 in the other three methods (Weighted Median, ML, and Weighted Mode) and consistent direction in Egger’s test were considered to have a significant causal relationship. We also reported results as suggestive causal relationships if they had a *p* < 0.05 but were above the Bonferroni-corrected threshold. Notably, we only considered results based on more than three shared SNPs [32]. The results were expressed as betas for the risk of sleep-related traits, corresponding to each standard deviation unit increase in the abundance of bacterial taxa and pathways, and vice versa for the betas on bacterial taxa and pathways per one standard deviation unit increase in sleep-related traits.

### 2.5. Sensitivity Analysis

To ascertain the robustness of the identified significant causal relationships, we conducted a series of sensitivity analyses. Cochran’s Q statistic was employed to evaluate heterogeneity among different SNPs [33]. A *p*-value greater than 0.05 in this test indicates an absence of significant heterogeneity among the instrumental variables used in our analysis. For assessing potential horizontal pleiotropy, we calculated the intercept of MR-Egger; a *p* > 0.05 suggests no evident horizontal pleiotropy among the SNPs in our MR analysis [34]. In addition, we utilized the MR-PRESSO global test to detect horizontal pleiotropy. This test compares the observed distance of all variants to the regression line (residual sum of squares) against the expected distance under the null hypothesis of no horizontal pleiotropy. The MR-PRESSO outlier test was also performed to identify specific horizontal pleiotropic outlier variants [35]. Furthermore, we conducted a ‘leave-one-out’ analysis, which involves sequentially excluding each SNP to assess its individual influence on the primary causal relationship. All MR analyses and sensitivity tests were performed using the TwoSampleMR (version 0.5.6), MR-PRESSO (version 1.0), and other relevant packages in R (version 4.2.0).

## 3. Results

### 3.1. Selection of the Instrumental Variables

Based on our instrumental variables (IVs) selection criteria, two taxa, species *Bacteroides* and species *Lachnospiraceae*, were excluded as they lacked SNPs below the threshold of 1.0 × 10^−5^. The number of IVs for 205 microbial taxa and 205 pathways ranged from 2 to 19, explaining 0.26–5.15% of the variance in their corresponding taxa and pathways. The lowest F statistic observed among these IVs was 19.51, signifying that all IVs possessed sufficient strength for the MR analysis of the 410 gut microbiotas and associated pathways. In the reverse MR analysis, the number of IVs varied significantly across different sleep-related traits: 46 for sleep duration, 51 for ease of getting up in the morning, and 105 for morningness, accounting for a variance between 0.48% and 1.36%. These three sleep-related traits are all continuous variables. However, we encountered limitations in identifying adequate IVs for the traits of insomnia, snoring, daytime dozing, and daytime napping. Comprehensive details on the selected IVs are available in Supplementary File S2: Tables S1 and S2.

### 3.2. Suggestive Causal Relationships between Microbial Taxa and Biological Pathways and Sleep-Related Traits

**Figure 2:**
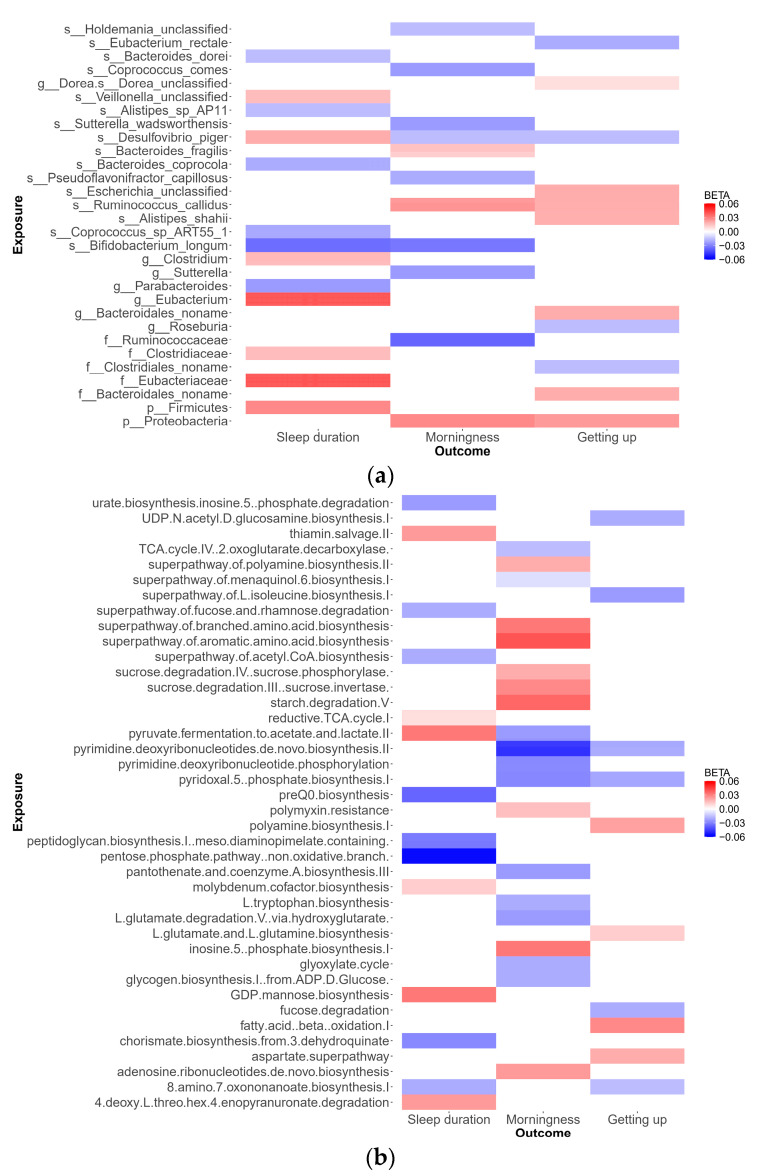
Suggestive causal relationships of microbial taxa and pathways on sleep-related traits: (**a**) Microbial taxa on sleep-related traits; (**b**) pathways on sleep-related traits.

As depicted in Figure 2, our analysis identified 79 suggestive causal effects of microbial taxa and pathways on sleep-related traits, each conforming at a P_IVW_ < 0.05. These effects spanned 70 microbial taxa and pathways and affected 3 distinct sleep-related traits, with betas ranging from −0.05 to 0.04. Among these 79 suggestive causal relationships, 44 were associated with microbial pathways. Of the 35 taxa identified, 21 were specific at the species level. Conversely, in the analysis of inverse suggestive causal relationships, we observed 45 pairs, with 20 related to pathways (Supplementary File S1: Figure S1). Among the remaining 25 pairs, 16 were specific at the species level, with betas ranging from −0.99 to 1.34.

*Desulfovibrio* emerged as a notable species, exhibiting suggestive causal relationships with all three sleep disorders studied. Specifically, the genetically determined abundance of *Desulfovibrio* showed a negative correlation with morningness (β −0.02; SE 0.009; P_IVW_ = 5.0 × 10^−2^) and ease of getting up in the morning (β −0.02; SE 0.007; P_IVW_ = 2.3 × 10^−2^), while being positively associated with increased sleep duration (β 0.02; SE 0.01; P_IVW_ = 2.4 × 10^−2^). These findings highlight the potential of metagenomic sequencing data to provide detailed resolution at the species level and emphasize the significant role of pathways in our study. Detailed data supporting these suggestive causal relationships are available in Supplementary File S2: Tables S3–S6.

### 3.3. Causality of Genetically Determined Microbial Taxa and Pathways on Sleep-Related Traits

**Figure 3:**
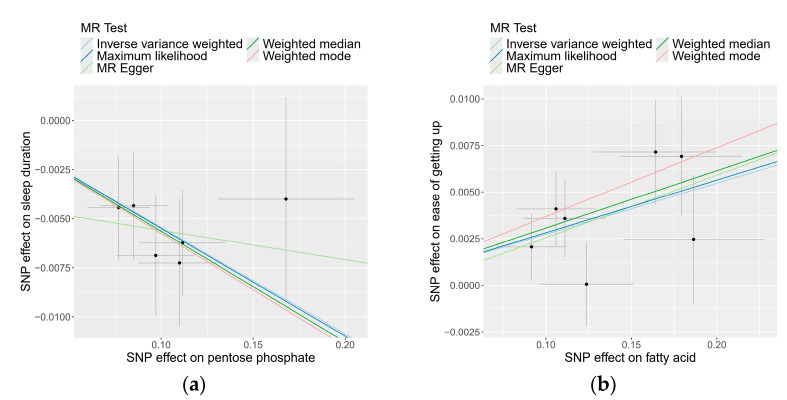
Scatter plots for the causal association of microbial taxa and pathways on sleep-related traits: (**a**) pentose phosphate pathway on sleep duration; (**b**) fatty acid pathway on ease of getting up in the morning.

Following the application of the Bonferroni correction, two causal relationships were identified that surpassed the threshold (P_IVW_ < 2.4 × 10^–4^). The IVW estimate revealed that a higher abundance of the pentose phosphate pathway decreases sleep duration (β −0.05; SE 0.012; *p* = 9.00 × 10^−6^) for each SD increase in pathway abundance. Additional methods corroborated this finding: the Weighted Median analysis (β −0.06; SE 0.016; *p* = 2.94 × 10^−4^), Weighted Mode analysis (β −0.06; SE 0.022; *p* = 4.74 × 10^−2^), and ML analysis (β −0.05; SE 0.013; *p* = 3.53 × 10^−5^) indicated consistent results (Table 1 and Figure 3). Additionally, the IVW estimate suggested that an increase in fatty acid levels has a positive causal effect on the ease of getting up in the morning (β 0.03; SE 0.007; *p* = 8.06 × 10^−5^). This was supported by the Weighted Median analysis (β 0.03; SE 0.010; *p* = 1.59 × 10^−3^), Weighted Mode analysis (β 0.04; SE 0.015; *p* = 4.30 × 10^−3^), and ML analysis (β 0.03; SE 0.007; *p* = 1.48 × 10^−4^), all aligning with the IVW findings. In both sets of causal relationships, the direction of effect estimated by MR-Egger was consistent with the results of the other four methods.

**Figure 4:**
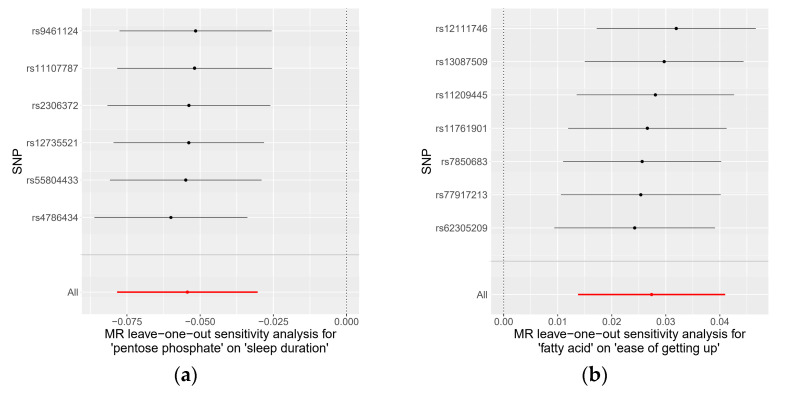
Leave-one-out plots for the causal association of microbial taxa and pathways on sleep-related traits: (**a**) pentose phosphate pathway on sleep duration; (**b**) fatty acid pathway on ease of getting up in the morning.

To evaluate the robustness of the causality for the two identified causal relationships, we conducted comprehensive sensitivity and pleiotropy analyses. For the pentose phosphate pathway on sleep duration, the funnel plot (Supplementary File S1: Figure S2) displayed a symmetrical distribution of SNPs, indicating no apparent horizontal pleiotropy or heterogeneity in our MR analysis. The Cochran’s Q statistic for the IVW method was 1.41 (*p* = 0.92), signifying no significant heterogeneity. Additionally, the MR-Egger regression revealed an insignificant intercept (intercept = −0.004, SE = 0.00586, *p* = 0.52), supporting the absence of horizontal pleiotropy. The MR-PRESSO global test (*p* = 0.95) and outlier test identified no outlier variants. Furthermore, the leave-one-out analysis confirmed that no individual SNP significantly influenced the results (Figure 4). Similarly, SNPs displayed no heterogeneity for the fatty acid pathway on ease of getting up in the morning, with a Cochran’s Q statistic of 4.51 (*p* = 0.61) in the IVW method. The MR-Egger regression (intercept = −0.0008, SE = 0.0035, *p* = 0.83), MR-PRESSO global test (*p* = 0.95), and outlier tests all indicated no evidence of horizontal pleiotropy or outliers. The leave-one-out analysis also demonstrated that the observed result was not driven by any individual SNPs. These sensitivity analyses collectively suggest that the causal effects of both the pentose phosphate and fatty acid pathways on their respective outcomes are robust and reliable. Detailed data on these sensitivity analyses are provided in Table 2.

## 4. Discussion

While previous research has explored the causal relationship between the gut microbiome and sleep phenotypes [25,36,37], many studies have predominantly focused on the genus level, limiting the exploration of specific species of gut microbes and their potential influence on sleep patterns. Our study advances this exploration by utilizing summary statistics of microbial taxa and pathways from the DMP cohort, which can allow identification to the species level and identify pathways. Through a two-sample MR analysis, we identified 79 suggestive causal effects of microbial taxa and pathways on sleep phenotypes, employing genetic variants as instrumental variables. Notably, eight gut microbiomes were linked to more than one sleep-related trait, indicating a multifaceted relationship. Conversely, our analysis revealed that four sleep-related traits exerted causal effects on 45 microbial taxa and pathways, with 3 of these taxa and pathways being influenced by more than one type of sleep-related trait. This underscores the complex interplay between sleep and the gut microbiome. Significantly, only two causal relationships surpassed the stringent criteria set by Bonferroni correction. These were the decrease in sleep duration due to the genetically determined relative abundance of the pentose phosphate pathway and the increase in ease of getting up in the morning associated with elevated fatty acid levels. The identification of these pathways highlights their substantial influence on sleep-related traits.

The pentose phosphate pathway (PPP) is a crucial glucose-metabolizing pathway with diverse physiological and pathological roles [38,39,40,41]. It is instrumental in generating biosynthetic precursors such as ribose 5-phosphate and nicotinamide adenine dinucleotide phosphate (NADPH) and in bolstering cellular defense mechanisms against oxidative stress. The pathway’s significance extends to various biological processes, including cell proliferation, differentiation, apoptosis, anti-infection responses, inflammation, tumorigenesis, and neurodegenerative diseases. Some observational studies have reported the association between PPP and sleep [42,43]. For instance, fructus gardeniae, known to alleviate anxiety symptoms caused by sleep deprivation, has been linked to the modulation of intestinal flora. In sleep-deprived mice, PPP was significantly enriched in the group treated with a low dose of gardeniae but not in those receiving a high dose [44]. This suggests a positive correlation between PPP enrichment and sleep deprivation, aligning with the findings of our study. Notably, research by Rey et al. [39] indicated that inhibiting PPP can alter the circadian rhythm of human cells. This alteration involves circadian transcription factors such as transcription factor BMAL1/CLOCK and the redox-sensitive transcription factor NRF2 [39]. We hypothesize that PPP influences sleep duration by affecting the expression of genes related to the circadian rhythm, offering one possible mechanism through which PPP reduces sleep time. Furthermore, the role of adenosine, a compound known to regulate sleep homeostasis [45], in conjunction with PPP’s consumption of adenosine, suggests another potential pathway through which PPP might impact sleep duration.

Numerous studies [46,47,48,49,50,51,52,53,54] have established correlations between fatty acids and sleep. For example, significant increases in non-esterified fatty acid (NEFA) levels are observed during restricted sleep periods [46]. Various fatty acids have been positively correlated with sleep quality [47], while short-chain fatty acids are known to affect sleep efficiency and latency [48]. Additionally, research has linked obstructive sleep apnea (OSA) with elevated free fatty acid levels [49], noting that OSA can increase free fatty acids and other metabolites during sleep, potentially leading to diabetes and cardiovascular diseases [50]. These findings indicate intricate interactions between fatty acids, sleep quality, and metabolic health. Moreover, a recent study has shown that hypertensive patients with OSA exhibit worse sleep parameters and altered electrolyte balances, suggesting that managing hypertension may further influence these metabolic interactions, impacting overall sleep quality and health outcomes [51]. Here, our study identifies a causal relationship between fatty acid pathways and the ease of getting up in the morning, thereby enriching the understanding of the interplay between sleep and fatty acids. Investigative efforts have been made to elucidate underlying mechanisms. For instance, fatty acid β-oxidation has been implicated in regulating θ oscillations during sleep [52], which is essential for sleep quality and memory consolidation. The association of fatty acid-binding proteins (FABPs) with fragmented sleep suggests that different components of fatty acid metabolism may variably affect sleep architecture [53]. Studies on mice with fatty acid amide hydrolase knockout exhibit enhanced slow-wave sleep [54], and consumption of short-chain fatty acids has been shown to decrease wakefulness and augment Non-Rapid Eye Movement (NREM) sleep [55], directly demonstrating the influence of fatty acids on sleep dynamics. We hypothesize that an increased abundance of short-chain fatty acids may reduce NREM sleep, facilitating easier awakening and rising in the morning. This mechanism might involve the regulation of FABP and the synthesis of sleep-promoting compounds like prostaglandin D2 [56], potentially impacting θ oscillations.

This study boasts several methodological strengths that enhance its validity and reliability. The first notable advantage is the choice of dataset. The GWAS summary data for seven sleep traits were sourced from a large-scale cohort comprising over 300,000 participants. This substantial sample size allowed the inclusion of a vast array of genetic variants associated with sleep-related traits, thereby boosting statistical power and minimizing heterogeneity that could arise from analyzing data across different studies. Moreover, the diverse sleep phenotypes analyzed in this study allowed for a more nuanced investigation of the associations between the gut microbiome and various aspects of sleep. For the gut microbiome, our study utilized the largest available GWAS dataset capable of identifying species and pathways, encompassing 7738 individuals. In contrast to many cohorts that depend on 16S rRNA measurements, our dataset facilitated an in-depth exploration of causal relationships at both the species level and in terms of bacterial pathway abundances. This approach underscores the importance of examining species abundance and pathway data in studying the gut microbiome’s impact on sleep. Additionally, the application of MR analysis represents another critical methodological advantage, providing less biased estimations of causal relationships free from confounding factors. The two-sample MR enables the analysis of data from distinct cohorts, thereby broadening the range of cohorts. The bidirectional MR approach enables the assessment of potential reverse causation. Moreover, the implementation of various MR methods and comprehensive sensitivity analyses further bolsters the reliability and robustness of our results. Lastly, efforts to minimize potential biases ensure the reliability of our findings. The GWAS summary data for exposure and outcome were derived from distinct, non-overlapping cohorts, thereby reducing the potential bias arising from sample overlap. Given that the datasets primarily consisted of individuals of European descent, the impact of population stratification has been minimized. These measures enhance the integrity of our research and ensure that our findings are robust.

While our study presents significant findings, it is essential to consider certain limitations. A major constraint is the reliance on the sample size of the GWAS. Despite using the largest GWAS dataset available that identifies gut microbiota at the species level, the sample size for the gut microbiota remains relatively modest. Therefore, it is necessary to increase the sample size to obtain more reliable IVs to explain the variation of the differences in taxa and pathway abundances. Another limitation arises from the SNP selection criteria. To acquire sufficient SNPs for the gut microbiome, the SNPs included in this study did not meet the conventional GWAS significance threshold (*p* < 5 × 10^–8^). Additionally, the genetic variants used explained only a small fraction of the gut microbiome; thus, they may not serve as precise proxies. Our understanding of the biological functions of the genetic instruments is still evolving, which introduces a potential risk of violating the independence and exclusion restriction assumptions of MR, particularly concerning pleiotropy. Also, our study’s findings are based on data from individuals of European descent and may not be generalizable to non-European populations. Lastly, while this study has identified two pathways with causal effects on sleep-related traits, these findings are primarily based on statistical analyses. Experimental research is required to elucidate the exact roles of these pathways in the pathogenesis of sleep traits and to validate our results further.

## 5. Conclusions

In conclusion, our two-sample bidirectional MR study has successfully delineated the potential influence of 79 microbial taxa and pathways on three specific sleep-related traits. Conversely, we identified 45 microbial taxa and pathways that appeared to be impacted by four different sleep phenotypes. Notably, our analysis revealed a robust effect of the pentose phosphate pathway on sleep duration and a significant influence of the fatty acid pathway on the ease of getting up in the morning. These findings illuminate the complex relationship between microbial pathways and sleep, underscoring how specific microbial factors influence various sleep-related traits. Further investigation, including randomized controlled trials, is required to validate these results and to fully understand the underlying mechanisms driving these associations.

## Figures and Tables

**Table 1:** MR results for the relationship between microbial pathways and sleep-related traits.

Method	Number of SNPs	F	β (SE)	*p*
Pentose phosphate pathway on sleep duration				
IVW	6	21.22	−0.05 (0.012)	9.00 × 10^−6^
Weighted median			−0.06 (0.016)	2.94 × 10^−4^
Weighted mode			−0.06 (0.022)	4.74 × 10^−2^
ML			−0.05 (0.013)	3.53 × 10^−5^
MR-Egger			−0.01 (0.057)	0.81
Fatty acid pathway on ease of getting up in the morning				
IVW	7	21.53	0.03 (0.007)	8.06 × 10^−5^
Weighted median			0.03 (0.010)	1.59 × 10^−3^
Weighted mode			0.04 (0.015)	4.30 × 10^−2^
ML			0.03 (0.007)	1.48 × 10^−4^
MR-Egger			0.03 (0.028)	0.28

**Table 2:** Heterogeneity and horizontal pleiotropy analyses between pathways and sleep-related traits.

Heterogeneity		Horizontal Pleiotropy			MR-PRESSO
IVW Q	IVW *p* Value	Egger Intercept	SE	*p* Value	Global Test *p* Value
Pentose phosphate pathway on sleep duration					
1.41	0.92	−0.004	0.0058	0.52	0.95
Fatty acid pathway on ease of getting up in the morning					
4.51	0.61	−0.0008	0.0035	0.83	0.70

## Data Availability

The original contributions presented in the study are included in the article/Supplementary Material, further inquiries can be directed to the corresponding author.

## Supplementary Materials

The following supporting information can be downloaded at: https://www.mdpi.com/article/10.3390/genes15060769/s1, Supplementary File S1: Figure S1: Suggestive causal relationships of sleep-related traits on microbial taxa and pathways: (**a**) sleep-related traits on microbial taxa; (**b**) sleep-related traits on pathways; Supplementary File S1: Figure S2: MR effect size for the causal association of microbial taxa and pathways on sleep-related traits: (**a**) pentose phosphate pathway on sleep duration; (**b**) fatty acid pathway on ease of getting up in the morning; Supplementary File S1: Figure S3: Funnel plots for the causal association of microbial taxa and pathways on sleep-related traits: (**a**) pentose phosphate pathway on sleep duration; (**b**) fatty acid pathway on ease of getting up in the morning; Supplementary File S2: Table S1: Instrumental variables used in MR analysis of 205 taxa and 205 pathways; Supplementary File S2: Table S2: Instrumental variables used in MR analysis of 3 sleep-related traits; Supplementary File S2: Table S3: Full result of MR estimates for the association of microbial taxa and pathways on sleep-related traits; Supplementary File S2: Table S4: Full result of MR estimates for the association of sleep-related traits on microbial taxa and pathways; Supplementary File S2: Table S5: 79 suggestive causal relationships of microbial taxa and pathways on sleep-related traits; Supplementary File S2: Table S6: 45 suggestive causal relationships of sleep-related traits on microbial taxa and pathways. Supplementary File S1: Table S7: GWAS summary statistics: source and description.
