# Supplementary material for: Causality Investigation between Gut Microbiome and Sleep-Related Traits: A Bidirectional Two-Sample Mendelian Randomization Study
Source: Genes (Basel). 2024 Jun 12;15(6):769. doi: 10.3390/genes15060769 (PMC11202894; doi:10.3390/genes15060769)
Supplement: Supplementary file 1 [file genes-15-00769-s001.zip › Supplementary File S1.pdf]

Table S7. GWAS summary statistics: source and description

Phenotypes	Consortium	Sample size	Download
207 taxa and 205 pathways	The Dutch Microbiome Project	7, 738	https://dutchmicrobiomeproject.molgeniscloud.org
Insomnia	UK Biobank	386, 533	https://ctg.cncr.nl/software/summary_statistics
Morningness	UK Biobank	345, 552	https://ctg.cncr.nl/software/summary_statistics
Sleep duration	UK Biobank	384, 317	https://ctg.cncr.nl/software/summary_statistics
Ease of getting up	UK Biobank	385, 949	https://ctg.cncr.nl/software/summary_statistics
Daytime napping	UK Biobank	386, 577	https://ctg.cncr.nl/software/summary_statistics
Daytime dozing	UK Biobank	386, 548	https://ctg.cncr.nl/software/summary_statistics
Snoring	UK Biobank	359, 916	https://ctg.cncr.nl/software/summary_statistics

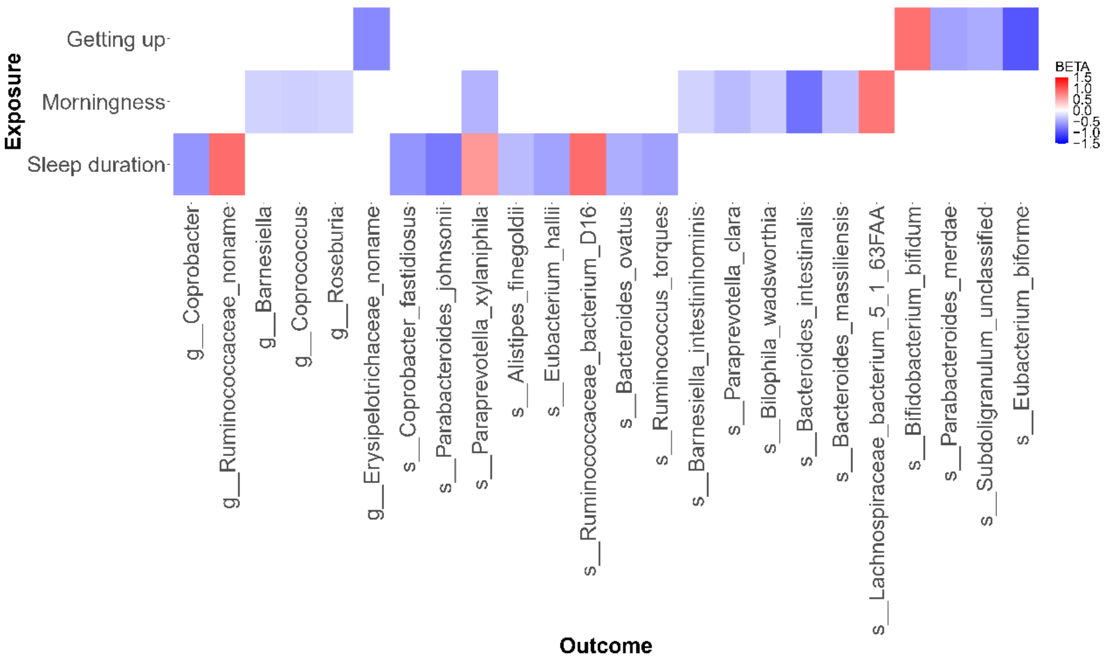

(a)

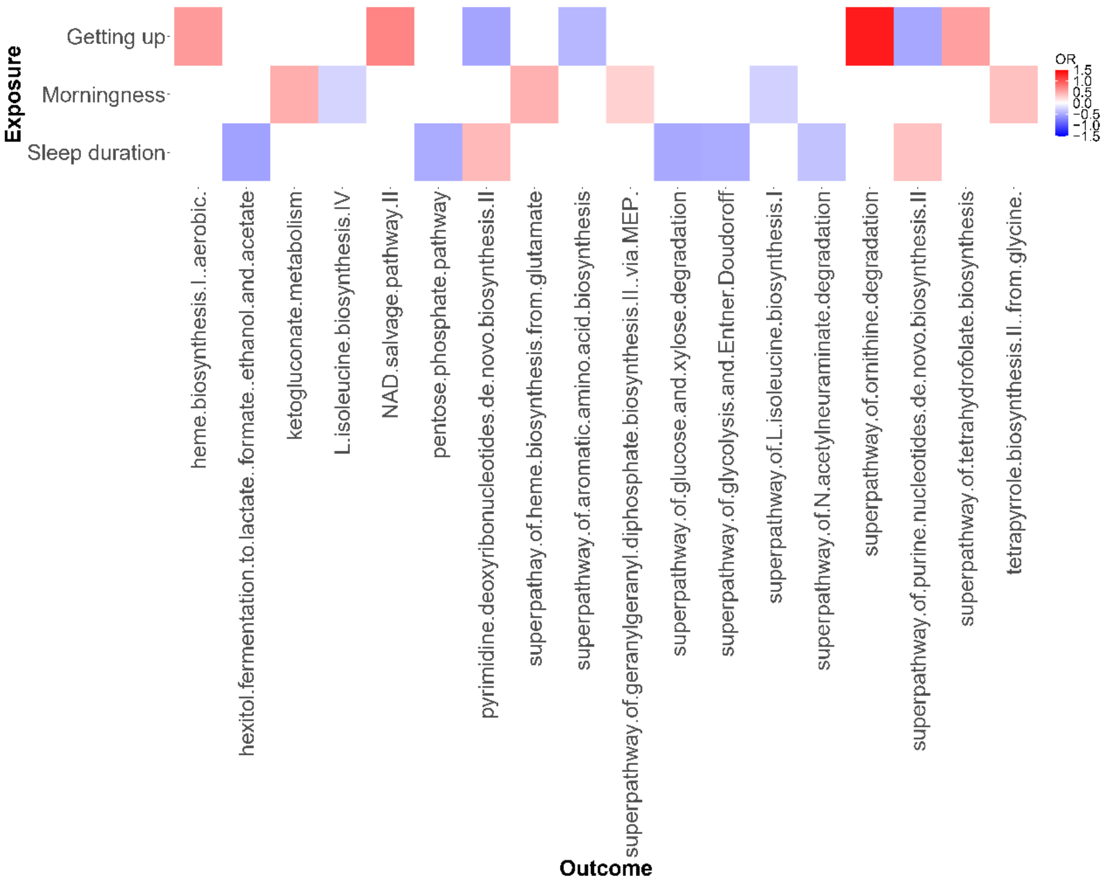

(b)

Figure S1. Suggestive causal relationships of sleep-related traits on microbial taxa and pathways: (a) Sleep-related traits on microbial taxa; (b) Sleep-related traits on pathways.

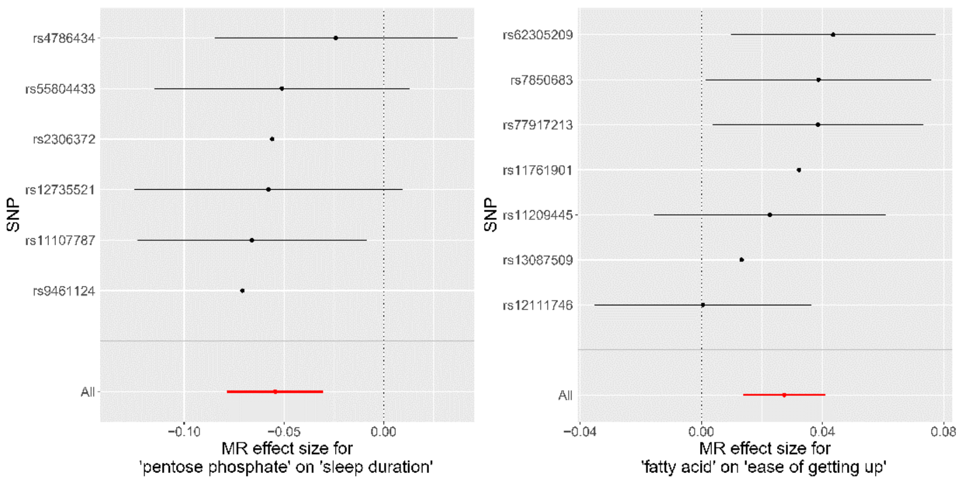

(a) (b)

Figure S2. MR effect size for the causal association of microbial taxa and pathways on sleep-related traits: (a) Pentose phosphate pathway on sleep duration; (b) Fatty acid pathway on ease of getting up in the morning.

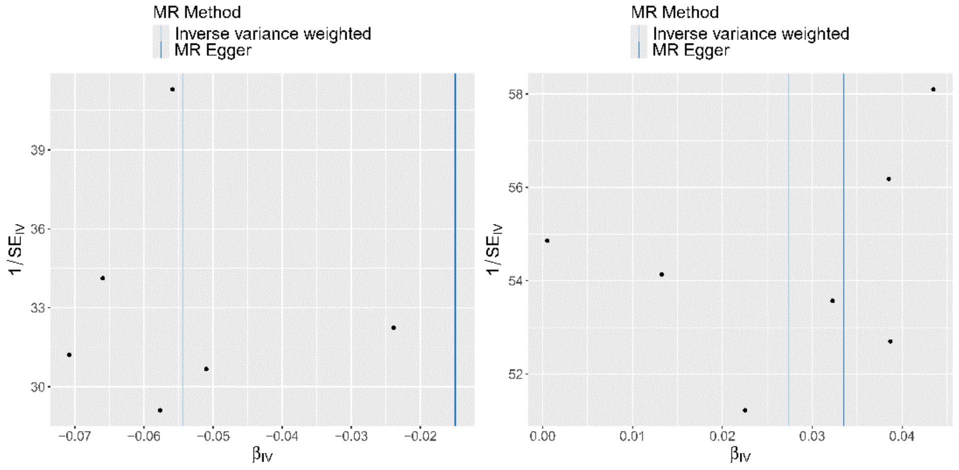

(a) (b)

Figure S3. Funnel plots for the causal association of microbial taxa and pathways on sleep-related traits: (a) Pentose phosphate pathway on sleep duration; (b) Fatty acid pathway on ease of getting up in the morning.
